# Promotion of Hyperthermic-Induced rDNA Hypercondensation in *Saccharomyces cerevisiae*

**DOI:** 10.1534/genetics.119.302994

**Published:** 2020-01-24

**Authors:** Donglai Shen, Robert V. Skibbens

**Affiliations:** Department of Biological Sciences, Lehigh University, Bethlehem, Pennsylvania 18015

**Keywords:** Cohesin, condensin, Hsp90/Hsp82/Hsc82, Chaperone, HMG, proteins, rDNA

## Abstract

Ribosome biogenesis is tightly regulated through stress-sensing pathways that impact genome stability, aging and senescence. In *Saccharomyces cerevisiae*, ribosomal RNAs are transcribed from rDNA located on the right arm of chromosome XII. Numerous studies reveal that rDNA decondenses into a puff-like structure during interphase, and condenses into a tight loop-like structure during mitosis. Intriguingly, a novel and additional mechanism of increased mitotic rDNA compaction (termed hypercondensation) was recently discovered that occurs in response to temperature stress (hyperthermic-induced) and is rapidly reversible. Here, we report that neither changes in condensin binding or release of DNA during mitosis, nor mutation of factors that regulate cohesin binding and release, appear to play a critical role in hyperthermic-induced rDNA hypercondensation. A candidate genetic approach revealed that deletion of either *HSP82* or *HSC82* (Hsp90 encoding heat shock paralogs) result in significantly reduced hyperthermic-induced rDNA hypercondensation. Intriguingly, Hsp inhibitors do not impact rDNA hypercondensation. In combination, these findings suggest that Hsp90 either stabilizes client proteins, which are sensitive to very transient thermic challenges, or directly promotes rDNA hypercondensation during preanaphase. Our findings further reveal that the high mobility group protein Hmo1 is a negative regulator of mitotic rDNA condensation, distinct from its role in promoting premature condensation of rDNA during interphase upon nutrient starvation.

PROTEIN synthesis in all organisms takes place in the highly conserved ribonucleoprotein complex—the ribosome. Ribosome biogenesis is thus directly related to cell growth and proliferation (Kief and Warner 1981). In eukaryotes, the nuclear compartment that assembles ribosomes (including rRNA synthesis, processing, and ribonucleoparticle assembly), is termed the nucleolus. rRNA arises from transcription of the rDNA locus that resides on the right arm of chromosome XII in the *Saccharomyces cerevisiae* yeast genome. This locus is ∼1–2 Mb and consists of ∼150 tandem repeats, each of which is 9.1 kb and encodes 5S, 5.8S, 25S, and 18S rRNAs (Nomura 2001; Spahn *et al.* 2001; Sirri *et al.* 2008).

Alterations in rDNA structure and function have implications far beyond the canonical roles of the nucleolus in rDNA transcription and ribosome biogenesis (Kobayashi and Sasaki 2017; Schöfer and Weipoltshammer 2018; Tiku and Antebi 2018). For instance, rDNA is the most highly represented gene in any eukaryote and also the most heavily transcribed locus (accounting for over 60% of the entire RNA pool) (Tomson *et al.* 2006). Due to this highly repetitive structure and active transcriptional status, rDNA is the most recombinogenic, and therefore mutagenic, site within the eukaryotic genome (Nomura 2001; Tomson *et al.* 2006; Kwan *et al.* 2016; Pal *et al.* 2018). The importance of maintaining rDNA locus stability is highlighted by the fact that DNA replication forks are programmed to stall within rDNA, precluding catastrophic head-on collision of replication and transcription complexes (Weitao *et al.* 2003; Shyian *et al.* 2016; Biswas *et al.* 2017). Furthermore, rDNA transcription rates, and even nucleolar size, are intimately coupled to changes in nutrient levels, revealing that rDNA plays a central role in responding to environmental cues (Li *et al.* 2006; Tsang *et al.* 2007; Wang *et al.* 2016). Disruption of rDNA transcription leads to ribosome biogenesis stress, and also inhibits Mdm2 function, resulting in cell cycle arrest, senescence, and apoptosis through p53-dependent pathways (Turi *et al.* 2018).

In yeast, changes in rDNA homeostasis impacts cellular aging and replicative lifespan in which extrachromosomal rDNA circles (ERCs), which arise through recombination, deplete the remaining genome of critical regulatory factors (Sinclair and Guarente 1997; Sinclair *et al.* 1997; Park *et al.* 1999; Shcheprova *et al.* 2008; Lewinska *et al.* 2014). Clinically, disruption of rDNA function in humans results in neurodegeneration, tumorigenesis, and severe developmental defects that include Treacher-Collins Syndrome, Blackfan Anemia, CHARGE Syndrome, and several others (Hallgren *et al.* 2014; Xu *et al.* 2014, 2017; Danilova and Gazda 2015; Wang and Lemos 2017; Udugama *et al.* 2018). Given that a rather surprisingly small percentage of nucleolar proteins function in ribosome biogenesis (Schöfer and Weipoltshammer 2018; Tiku and Antebi 2018), it becomes critical to explore the regulatory mechanisms through which rDNA responds to the many challenges imposed on the cell to ensure proper development and cell cycle regulation.

rDNA structure is tightly regulated through the cell cycle. In budding yeast, rDNA forms a diffuse puff-like structure during G1 phase that coalesces into a tight loop-like structure during mitosis (Guacci *et al.* 1994, 1997). The importance of these architectural changes is highlighted by the development of numerous strategies that include FISH, GFP-tagged rDNA binding proteins, and a streamlined intercalating-dye method that now provides for rapid and efficient quantification of rDNA condensation (Guacci *et al.* 1994, 1997; Lavoie *et al.* 2002; Lavoie *et al.* 2004; D’Ambrosio *et al.* 2008; Lopez-Serra *et al.* 2013; Tong and Skibbens 2015; Shen and Skibbens 2017a). To date, these condensation assays have helped elucidate the role of highly conserved cohesin and condensin complexes in regulating rDNA architecture. This is due to the fact that mutations in every cohesin and condensin subunit tested, or mutation of cohesion regulators such as the cohesin loader Scc2-Scc4 and cohesin acetyltransferase Eco1/Ctf7 (herein Eco1), produce profound impacts on condensation such that rDNA fails to compact during mitosis and appears instead as diffuse puff-like structures (Skibbens *et al.* 1999; Tóth *et al.* 1999; Kueng *et al.* 2006; D’Ambrosio *et al.* 2008; Hirano 2012; Lopez-Serra *et al.* 2013; Tong and Skibbens 2015). In addition to appropriate condensation reactions that occur during mitosis, the rDNA locus can also condense during G1 phase in response to nutrient starvation or rapamycin treatment. This premature rDNA condensation, which includes nucleolar contraction, requires *de novo* recruitment of condensin and the high mobility group protein Hmo1 (Tsang *et al.* 2007; Wang *et al.* 2016).

Despite the intense focus on yeast rDNA architecture over the last two decades (Guacci *et al.* 1994; Castaño *et al.* 1996; Freeman *et al.* 2000; Lavoie *et al.* 2004; Sullivan *et al.* 2004; Gard *et al.* 2009; Guacci and Koshland, 2012; Xu *et al.* 2017), an additional rDNA state was only recently discovered in which mitotic cells induce a hypercondensed rDNA state (dramatic shortening of the rDNA loop length) in response to elevated temperature (Shen and Skibbens 2017a; Matos-Perdomo and Machín 2018a). This hyperthermic-induced rDNA hypercondensation is both rapidly induced and reversible. Intriguingly, rDNA hypercondensation is also inducible by numerous cell stressors (rapamycin exposure, oxidative stress, nitrogen starvation, and caloric restriction) that inhibit the TORC1 pathway (Matos-Perdomo and Machín 2018a,b). The extent to which hyperthermic-induced rDNA hypercondensation is predicated on changes in either cohesin or condensin binding or release from DNA, however, remains unknown. Here, we find that, unlike the changes in either cohesin or condensin dynamics required for mitotic condensation, hyperthermic-induced rDNA hypercondensation occurs in the absence of altered levels on rDNA of either condensin or inactivation of factors that regulate cohesin binding/dissociation. Instead, we find that mutation of heat shock/chaperone Hsp90 family members Hsp82 and Hsc82 result in significantly reduced rDNA hypercondensation. Our results further identify Hmo1 as a negative regulator of mitotic rDNA condensation, in opposition to its role in rDNA premature-condensation that occurs during interphase upon nutrient starvation (Tsang *et al.* 2007; Wang *et al.* 2016).

## Materials and Methods

### Yeast strains and strain construction

*S. cerevisiae* genes and strains used in this study are listed in Table 1 (prioritized list of heat shock/chaperone encoding genes) and Table 2 (all yeast strains used in this study). Primers used to verify gene deletions within the Knockout collection are available upon request.

**Table 1:** Prioritized list of heat shock/chaperone encoding genes obtained from iterative GO terms searches and that represent a diverse set of cellular responses to elevated temperature

Common	Systematic	Descriptor	Reference
*FOB1*	YDR110W	rDNA replication fork barrier	SGD
*HIT1*	YJR055W	snoRNP assembly factor	SGD
*HMO1*	YDR174W	High mobility group factor	SGD
*HSP82*	YPL240C	Hsp90 chaperone	SGD
*ISW1*	YBR245C	Imitation-switch chromatin remodelers	SGD
*MSN2*	YMR037C	Stress-responsive transcriptional activator	SGD
*MSN4*	YKL062W	Stress-responsive transcriptional activator	SGD
*SIR2*	YDL042C	NAD+ dependent histone deacetylase	SGD
*SSA1*	YAL005C	ATPase member of HSP70 family	SGD
*TOP1*	YOL006C	Topoisomerase I	SGD

**Table 2:** Yeast strains used in this study

Strain name	Genotype	Reference
**YPH499**	*MATa*; *S288C*	Sikorski and Hieter (1989)
**YBS1039**	*MATa*; *w303*	Shen and Skibbens (2017a)
**BY4741**	*MATa*; *BY4741*	Brachmann *et al.* (1998)
**YBS1141**	*MATa*; *chl1*::*KAN*; *S288C*	Skibbens (2004)
**YBS2037**	*MATa*; *rad61*::*URA*; *w303*	Tong and Skibbens (2014)
**YMM511**	*MATa*; *scc2-4*; *can1-100*; *w303*	Maradeo *et al.* (2010)
**YBS3036**	*MATa*; *SMC2:3HA:KanMX6*; *w303*	Shen and Skibbens (2017b)
**YBS3047**	*MATa*; *hmo1*::*KanMX6*; *isolates1*	For this study
**YBS3048**	*MATa*; *hmo1*::*KanMX6*; *isolates2*	For this study
**YBS3049**	*MATa*; *hmo1*::*KanMX6*; *isolates3*	For this study
**YBS1129**	*Chl1:13Myc*	Skibbens (2004)
**YDS200**	*MATa*; *fob1*::*KanMX6*	Winzeler *et al.* (1999)
**YDS201**	*MATa*; *hit1*::*KanMX6*	Winzeler *et al.* (1999)
**YDS202**	*Diploid*; *hmo1*::*KanMX6*	Winzeler *et al.*, 1999
**YDS203**	*MATa*; *hsp82*::*KanMX6*	Winzeler *et al.* (1999)
**YDS204**	*MATa*; *isw1*::*KanMX6*	Winzeler *et al.* (1999)
**YDS205**	*MATa*; *msn2*::*KanMX6*	Winzeler *et al.* (1999)
**YDS206**	*MATa*; *msn4*::*KanMX6*	Winzeler *et al.* 1999
**YDS207**	*MATa*; *sir2*::*KanMX6*	Winzeler *et al.* (1999)
**YDS208**	*MATa*; *ssa1*::*KanMX6*	Winzeler *et al.* (1999)
**YDS209**	*MATa*; *top1*::*KanMX6*	Winzeler *et al.* (1999)
**YDS210**	*MATa*; *hsc82*::*KanMX6*	Winzeler *et al.* (1999)

### rDNA condensation assay

A streamlined condensation assay is adapted from a published FISH protocol (Guacci *et al.* 1997; Shen and Skibbens 2017a). Briefly, cells were arrested at preanaphase and fixed by paraformaldehyde for 2 hr at 23°. Cells were washed with distilled water and resuspended in spheroplast buffer (1 M sorbitol, 20 mM KPO_4_, pH 7.4), then spheroplasted by adding beta-mercaptoethanol and Zymolyase T100 and incubating for 1 hr at 23°. Resulting cells were added to poly-l-lysine coat slides, treated with 0.5% Triton X-100, 0.5% SDS, and dehydrated in 3:1 methanol:acetic acid. Slides were stored at 4° until completely dry, then cells were treated with RNase in 2X SSC buffer (0.3 M NaCl, 30 mM sodium citrate, pH 7.0), dehydrated and denatured at 72° following cold ethanol wash. DNA mass was detected by DAPI staining and assayed under a microscope. Cell cycle progression was confirmed by detection of DNA content using flow cytometry as described (Tong and Skibbens 2015).

### Chromatin immunoprecipitation and ChIP primers

Chromatin immunoprecipitation (ChIP) was performed as previously described (Rudra and Skibbens 2012), with the following modifications. Cells were cultured to log phase with OD600 1.0–1.2, then incubated at 23° in rich YPD medium supplemented with alpha-factor for 2.5 hr. The resulting cells were collected, washed, and then resuspended in fresh YPD supplemented with nocodazole, incubated at 23° or 37° for 3 hr, and then fixed in 1% formaldehyde for 20 min. Cells were then harvested, spheroplasted, and lysed. Cells lysates were sonicated on ice for six cycles of 10 sec. The suspension was centrifuged and diluted 1:10. The diluted suspension was then centrifuged and the supernatant was collected as the chromatin solution. Smc2 enrichment was obtained by incubating chromatin solution with EZ-View Red Anti-HA affinity matrix (Sigma) overnight at 4°; the background control was obtained in a similar manner by incubating the same batch of chromatin solution (isogenic strain expressing Smc2-HA) with EZ-View Red Anti-Myc affinity matrix (Sigma, used as beads only control) overnight at 4°. Beads were collected by centrifugation, washed, and the remaining bead-bound proteins harvested using 1% SDS; 0.1 M NaHCO3. DNA-protein crosslinks were reversed in 5 M NaCl for 4 hr at 65°. DNA precipitation from the resulting lysate was performed by overnight incubation at −20° in 70% ethanol. Precipitates were extracted in series using 25:24:1 phenol:chloroform:isoamylalcohol and pure chloroform prior to reprecipitation of DNA overnight at −20° in 70% ethanol. DNA was resuspended in TE buffer and analyzed by PCR using the rDNA primers previously described (Johzuka and Horiuchi 2009; Thattikota *et al.* 2018). PCR products were resolved using 1% agarose gels, and histograms of pixel densities quantified in Photoshop. Smc2 enrichment was calculated as the ratio of HA pull down all over total chromatin input.

### Statistical analyses

Tukey HSD one way ANOVA tests were used to assess statistical significance (*P* < 0.05).

### Data availability

Strains and plasmids are available upon request. The authors affirm that all data necessary for confirming the conclusions of the article are present within the article, figures, tables and in supplemental information available at the following URL (https://doi.org/10.25386/genetics.10283972). Supplemental material available at figshare: https://doi.org/10.25386/genetics.10283972.

## Results

### Hyperthermic-induced rDNA hypercondensation occurs in the absence of new cohesin deposition (Scc2 inactivation) and release (Rad61 deletion)

**Figure 1:**
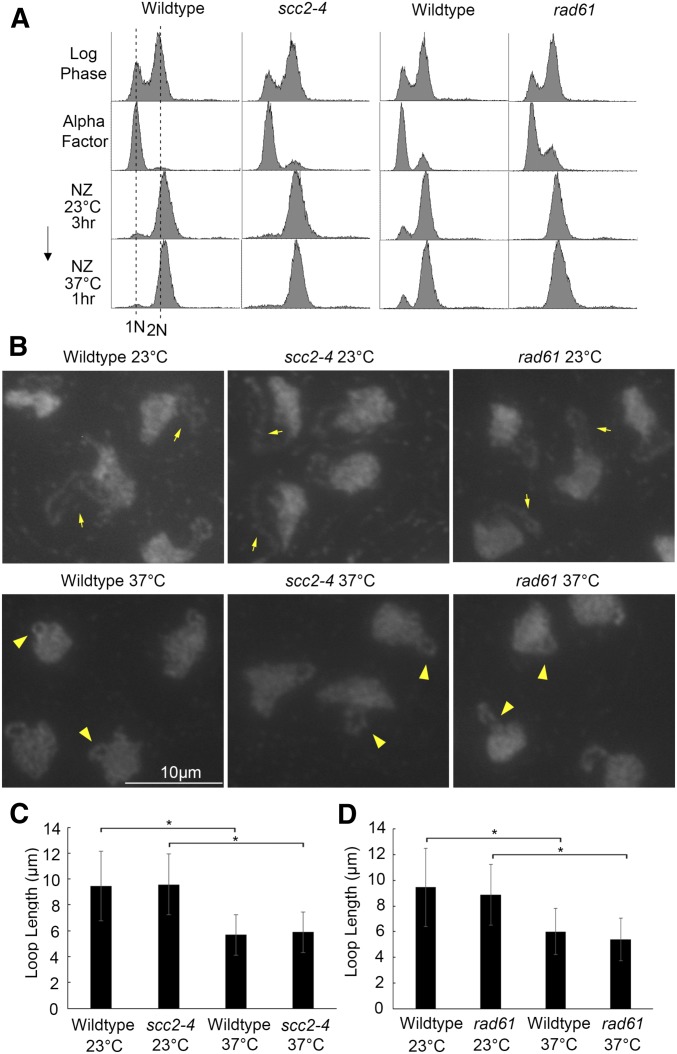
Cohesin deposition and/or release are not required for hyperthermic-induced rDNA hypercondensation. (A) Flow cytometer data documents DNA content of wild type (YBS1039), *scc2**-4* mutant cells (YMM511), and *rad61* null cells (YBS2037) throughout the experiment. Wild-type and *scc2**-4* mutant cells were processed separately from wild-type and *rad61* null cells. Cells were maintained in nocodazole for 3 hr at 23°, post-alpha factor arrest, followed by an additional 1 hr incubation at 37°. (B) Chromosomal mass and rDNA loop structures detected using DAPI. Yellow arrows indicate long rDNA loops. Yellow arrowheads indicate short rDNA loops. All field of views are shown at equal magnification. (C) Quantification of the loop length of condensed rDNA in wild-type and *scc2**-4* mutant cells. Data shown was obtained from three biological replicates. For wild type *vs.* *scc2**-4* mutant, the results reflect *n* values of 228 of wild-type cells at 23°, 235 for *scc2**-4* cells at 23°, 214 for wild-type cells at 37°, and 201 for *scc2**-4* cells at 37°. (D) Quantification of loop lengths of condensed rDNA in wild-type and *rad61* null cells. For wild-type *vs.* *rad61* null mutant, the results reflect n values of 235 for wildtype cells at 23°, 214 for *rad61* cells at 23°, 157 for wildtype cells at 37°, and 151 for *rad61* cells at 37°. Statistical analysis was performed using a Tukey HSD one way ANOVA. *P*-value = 0.721 indicates that there is no significant difference between the average loop lengths of wild-type cells (5.67 μm) and *scc2**-4* mutant cells (5.89 μm) at 37° in (C). *P*-value = 0.103 indicates there is no significant difference between wild-type cells (6.02 μm) and *rad61* null cells (5.40 μm) at 37° in (D). Statistically significant differences (*) are based on *P* < 0.05.

Wild-type cells shifted to an elevated temperature during mitosis exhibit rDNA hypercondensation (Shen and Skibbens 2017a; Matos-Perdomo and Machín 2018a), but the structural basis for this dramatic change in chromatin structure remains unknown. Cohesins play a critical role in chromosome condensation, including across the rDNA locus, such that mutation in genes that encode either cohesin subunits (Mcd1/Scc1, Pds5, or Scc3) or regulators (Eco1 or Scc2) all result in severe rDNA condensation defects (Guacci *et al.* 1997; Skibbens *et al.* 1999; Hartman *et al.* 2000; D’Ambrosio *et al.* 2008; Guacci and Koshland 2012; Orgil *et al.* 2015; Tong and Skibbens 2015; Woodman *et al.* 2015). These observations formally suggest that *de novo* cohesin deposition during mitosis may play a critical role in hyperthermic-induced rDNA hypercondensation, in contrast to the decondensation of rDNA into “puffs” that occurs upon either cohesin inactivation or dissociation (Guacci *et al.* 1997; Ciosk *et al.* 2000; Shen and Skibbens 2017a). Here, we test whether *de novo* cohesin deposition promotes hyperthermic-induced rDNA hypercondensation by the rapid inactivation (via the temperature-sensitive **scc2***-4* allele) of the Scc2,4 heterocomplex, which is required for cohesin deposition onto DNA (Ciosk *et al.* 2000; Watrin *et al.* 2006). Log phase cultures of wild-type and *scc2**-4* mutant cells were synchronized in G1 at 23° using rich medium supplemented with alpha factor, washed, and then arrested in preanaphase at 23° (permissive for *scc2**-4* cells) by incubation in medium supplemented with nocodazole. The resulting cultures were then shifted to 37° (nonpermissive for *scc2**-4* cells) for 1 hr while maintaining the preanaphase arrest. Cell cycle progression from log phase into mitosis was confirmed by flow cytometry (Figure 1A). As expected, mitotic wildtype cells maintained at 23° contained long rDNA loops while the rDNA of mitotic cells shifted to 37° during the final hour of incubation hypercondensed into very short loops (Figure 1B), consistent with prior findings (Shen and Skibbens 2017a; Matos-Perdomo and Machín 2018a). Previous analyses of these cells revealed that only a fraction of *scc2**-4* mutant cells contain condensed rDNA loci at 23°, a level that is retained after shifting to 37° during the final hour of incubation (Shen and Skibbens 2017b). We thus limited our current measurements to the fraction of cells in which rDNA loops were tightly cohered and condensed into discrete loops (Figure 1B). The results show that *scc2**-4* cells contain long rDNA loops at 23°. Importantly, rDNA in *scc2**-4* mutant cells fully hypercondense into very short loops after incubation at 37° for 1 hr (Figure 1, B and C). We confirmed that this *scc2**-4* mutant strain is indeed temperature sensitive, and defective in cohesin deposition onto chromatin (Shen and Skibbens 2017b). Thus, temperature-induced rDNA hypercondensation during mitosis occurs in the absence of *de novo* cohesin deposition.

Might rDNA hypercondensation result from cohesin dissociation? For instance, wild-type cells exhibit faster growth kinetics at 37°, despite containing hypercondensed rDNA (Shen and Skibbens 2017a; Matos-Perdomo and Machín 2018a). To accommodate the increase in rDNA transcription required for this faster rate of cell growth, cohesin dissociation from the rDNA loop (herein referred to as the longitudinal axis) might enable formation of lateral loops (orthogonal to the longitudinal rDNA axis) that are more accessible to the transcriptional machinery (Shen and Skibbens 2017a). In this model, we posit that cellular responses to stressors, such as heat, likely involves a pool of dynamic cohesins (*i.e.*, nonacetylated), which would allow for cohesin dissociation sufficient to induce hypercondensation, but not chromosome decondensation or loss of sister chromatid cohesion (Shen and Skibbens 2017a). To test whether short longitudinal rDNA loops (interpreted as hypercondensation) occur due to cohesin removal, we turned to the cohesin destabilizer Rad61/WAPL (Vernì *et al.* 2000; Game *et al.* 2003; Kueng *et al.* 2006; Sutani *et al.* 2009; Lopez-Serra *et al.* 2013). Cohesin-dissociation activity exhibited by Rad61/WAPL persists throughout the cell cycle, and, when induced to high levels specifically during mitosis, can result in loss of sister chromatid cohesion (Ström *et al.* 2007; Unal *et al.* 2007; Heidinger-Pauli *et al.* 2009; Terret *et al.* 2009; Lopez-Serra *et al.* 2013; Eser *et al.* 2017; Haarhuis *et al.* 2017; Wutz *et al.* 2017; Dauban *et al.* 2019). Log phase wildtype and *rad61* null cells were treated as described earlier to achieve sequential G1 and preanaphase synchronizations at 23° before shifting to 37° for 1 hr, while maintaining the mitotic arrest (Figure 1A). *rad61* null cells condensed the rDNA into extended discrete loops at 23°, similar to both wild-type and *scc2**-4* mutant cells. Moreover, *rad61* null cells were fully competent to hypercondense the rDNA into very short loops upon shifting to 37° for 1 hr (Figure 1, B and D). These results reveal that rDNA hypercondensation (longitudinal shortening) occurs in the absence of Rad61-dependent cohesin release from rDNA. In combination, these results reveal that mitotic hyperthermic-induced rDNA hypercondensation occurs independent of both Scc2-dependent deposition of new cohesins and Rad61-dependent release of pre-existing and dynamic cohesins.

Chl1 DNA helicase is a positive regulator of both sister chromatid cohesion and chromosome condensation in that Chl1 promotes both Scc2 and cohesin binding to DNA (Mayer *et al.* 2004; Skibbens 2004; Inoue *et al.* 2007; Xu *et al.* 2007; Laha *et al.* 2011; Rudra and Skibbens 2012; Borges *et al.* 2013; Samora *et al.* 2016; Shen and Skibbens 2017b), providing a further opportunity to assess whether cohesin deposition/release are involved in hyperthermic-induced rDNA hypercondensation. We previously reported analyses of rDNA loop lengths in wildtype cells (adapted from Figure 1E in Shen and Skibbens 2017a), but at that time did not include quantification of rDNA loop lengths in *chl1* mutant cells that were simultaneously assessed. As previously described, wild-type and *chl1* deletion cells were synchronized in G1 at 23°, then cultures were divided and released into either 23° or 37° medium supplemented with nocodazole to arrest cells in preanaphase. Cell cycle progression and arrests were confirmed using flow cytometry (Supplemental Material, Figure S1A). Our results reveal that *chl1* null cells, shifted to 37°, were fully competent to hypercondense the rDNA into very short loops, similar to wild-type cells and in contrast to the elongated rDNA loops present at 23° in both wild-type and *chl1* mutant cells (Figure S1, B and C and Shen and Skibbens 2017a). Thus, hyperthermic-induced rDNA hypercondensation occurs in the absence of Chl1, consistent with a mechanism independent of Scc2-mediated cohesin loading.

### Condensin deposition and/or release are not required for hyperthermic-induced rDNA hypercondensation

**Figure 2:**
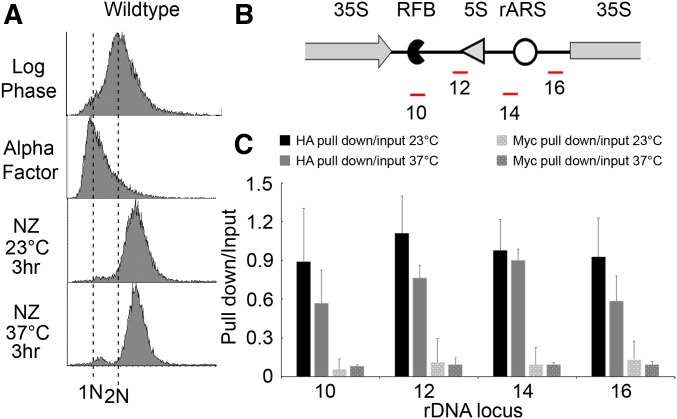
Condensin deposition and/or release appear independent of hyperthermic-induced rDNA hypercondensation. (A) Flow cytometer data documents DNA content throughout the experiment. Wild-type cells (YBS3036) were maintained in nocodazole for 3 hr at 23° or 37° postalpha factor arrest. (B) Schematic indicates the location of four ChIP primer sets (in red) that reside within the interval region flanked by two rDNA repeats. RFB, Replication Fork Barrier; rARS, rDNA Autonomously Replicating Sequence. (C) Quantification of ChIP using the primer pairs shown in (B) to assess Smc2 enrichment (HA pull down/input) in mitotic wild-type cells maintained at 23° *vs.* 37°. Data shown was obtained from three biological replicates. Error bars represent SD of each sample. Statistical analysis was performed using Tukey HSD one way ANOVA. *P*-value = 0.481, 0.185, 0.899, and 0.215 for primer sets 10, 12, 14, and 16 respectively, and indicate that there is no significant difference between the Smc2-HA enrichment of wild-type cells arrested at 23° and 37°. Note that combining data obtained from the four sites, and then comparing the average Smc2 enrichment from three independent biological replicates at 23 degree to 37 degree using Tukey HSD one way ANOVA, produces a *P*-value of 0.29. This further indicates that, by testing for trends across the rDNA in aggregate, condensin deposition and/or release are not required for hyperthermic-induced rDNA hypercondensation. Statistically significant differences (*) are based on *P* < 0.05. *P*-values of overall HA pull down/input, *vs.* Myc pull down/input, are statistically significant (0.001 at 23°, and 0.01 at 37°) for both temperatures.

Mitotic chromosome condensation requires condensin, in addition to cohesin, such that condensin mutants exhibit severe condensation defects along the rDNA locus (Strunnikov *et al.* 1995; Freeman *et al.* 2000; Lavoie *et al.* 2002; Lavoie *et al.* 2004; D’Ambrosio *et al.* 2008). Unlike the cohesin complex, there is no known loading complex that promotes condensin deposition onto chromosomes (Hirano 2012; Ganji *et al.* 2018). Thus, to assess whether condensin deposition is required for hyperthermic-induced rDNA hypercondensation, we directly tested for hyperthermic-induced changes in condensin binding to rDNA using ChIP. Wild-type cells expressing HA-tagged Smc2 were synchronized in G1 at 23°, then divided into two, with aliquots released into fresh medium at either 23° or 37° supplemented with nocodazole to arrest cells in preanaphase (Figure 2A). Protein–DNA complexes were cross-linked using formaldehyde, followed by cell lysis and sonication to shear the DNA. Chromatin complexes containing Smc2 were immunoprecipitated (HA pull down), cross-links reversed, and condensin enrichment quantified from PCR using four well-documented condensin-binding sites within the rDNA locus (Figure 2B) (Johzuka and Horiuchi 2009; Thattikota *et al.* 2018). Background control was obtained by immunoprecipitation using affinity matrix targeting Myc, which is not expressed in the cell (Myc pull down). The ratio of HA pull down over input is significantly higher than the Myc pull down over input. Thus, HA pull down/input specifically represents Smc2 enrichment (Figure 2C). The results, averaged across all four sites and based on three independent biological replicates (at 23° *vs.* 37°), reveal no change in Smc2 enrichment (*P*-value = 0.29), despite dramatic changes in rDNA structure. Even on a site-by-site analyses, the results suggest that Smc2 levels do not increase during rDNA hypercondensation but instead remain relatively unchanged at 23° compared with 37° (Figure 2C). Thus, hyperthermic-induced rDNA hypercondensation occurs independent of both condensin deposition and dissociation.

A recent study suggests that rDNA condensation and nucleolar compaction progress to a maximum state during early anaphase (de los Santos-Velazquez *et al.* 2017). While this rDNA condensation appears separate from the significant decrease in longitudinal rDNA loop lengths that occur in response to heat-stress preanaphase (Shen and Skibbens 2017a; Matos-Perdomo and Machín 2018a), we decided to augment our arrest strategy to ensure that cells are not escaping the nocodazole-induced mitotic arrest. Cdc23 is an essential component of the Anaphase Promoting Complex (Hartwell *et al.* 1973; Lamb *et al.* 1994; Irniger *et al.* 1995). *cdc23**-1* mutant strains were synchronized in G1 (alpha factor) and then released into 37° (restrictive for *cdc23* alleles) medium supplemented with or without DMSO or nocodazole. DNA profiles confirm the efficacy of *cdc23* mutant protein inactivation in that 2N DNA profiles were obtained at the end of the 3 hr temperature incubation at 37° regardless of the presence or absence of nocodazole or DMSO (Figure S2A). Nuclei in APC mutants cells, arrested preanaphase in the absence of nocodazole, experience mitotic forces via kinetochore microtubules and spindles. We have observed a large population (∼70%) of distort chromatin that result in indiscernible rDNA loop structures (Figure S2B, red arrows). Prior findings similarly reported that the strategies used to arrest cells preanaphase impact rDNA architectures (Guacci *et al.* 1994). More importantly, *cdc23*-*1* cells exhibited highly hypercondensed rDNA under hyperthermic conditions (driving both rDNA axial shortening and APC inactivation) with or without nocodazole (Figure S2B, yellow arrows). These results confirm that rDNA hypercondensation can be induced prior to anaphase solely by increased temperature and is not a byproduct of nocodazole treatment.

### Hyperthermic-induced rDNA hypercondensation is separate from several activities that impact rDNA regulation

The surprising findings that changes in rDNA association of cohesin and condensin do not appear to contribute to hyperthermic-induced rDNA hypercondensation suggested that a novel mechanism must exist by which cells regulate rDNA structure in response to thermic stress. We thus turned to heat-shock pathways through which cells appropriately respond to elevated temperatures (Verghese *et al.* 2012), even though no evidence to date directly implicates heat shock proteins/chaperones (HSP/C) either in mitotic rDNA condensation or hyperthermic-induced hypercondensation. To generate a candidate list, we first took a bioinformatics approach and queried the Saccharomyces Genome Database (SGD) GO term database using an iterative process in which each search contained unique combinations of any two of several terms (Response to heat; Nucleolus; Chromatin binding; Regulation of DNA metabolic process, etc.). We cross-referenced the resulting lists to identify candidates that occur in high frequency, and then selected those in which mutations are readily obtainable from a prototrophic deletion collection (Winzeler *et al.* 1999; Giaever and Nislow 2014; VanderSluis *et al.* 2014). We finally prioritized 10 genes that provide the most extensive coverage of independent heat shock/chaperone pathways (Table 1).

**Figure 3:**
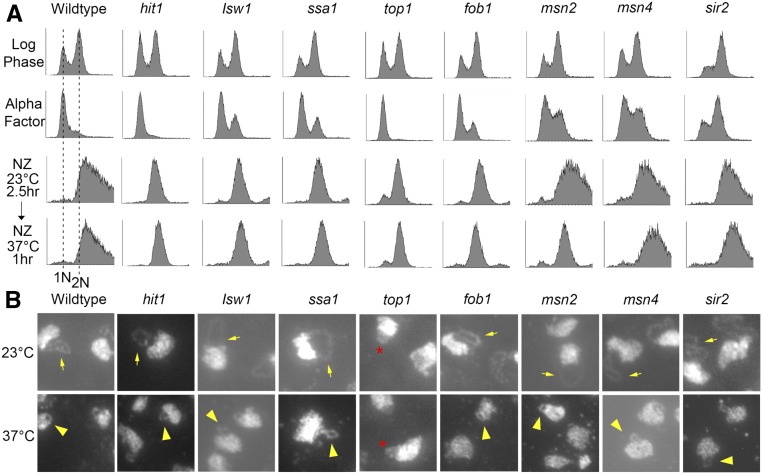
Hyperthermic-induced rDNA hypercondensation is separate from several activities that impact rDNA regulation. (A) Flow cytometer data documents DNA contents of log phase cells (wild type and indicated HSP/C mutant strains) synchronized in G1 (Alpha Factor), then subsequently arrested in preanaphase (NZ) at 23° for 2.5 hr before shifting to 37° for 1 hr. (B) Chromosomal mass and rDNA loop structures detected using DAPI. Yellow arrows indicate long rDNA loops. Yellow arrowheads indicate short rDNA loops. Red star indicates the decondensed rDNA puff observed in *top1* null mutant.

Wild-type and all 10 heat shock protein/chaperone (HSP/C) null cells were sequentially synchronized in G1 and preanaphase as described above, before shifting the resulting mitotic cells to 37° for an additional 1 hr while maintaining the mitotic arrest. Cell cycle synchronizations and progression for each strain were monitored using flow cytometry (Figure 3A). As expected, rDNA in wildtype cells exhibited significantly hypercondensed rDNA loops after shifting to 37° for 1 hr (Figure 3B). Not surprisingly, the bulk of the HSP/C candidates (*msn2*, *msn4*, *ssa1*, *sir2*, *isw1*, *hit1*, and *fob1*) exhibited both normal mitotic rDNA condensation at 23° and hypercondensation at 37° (Figure 3B). Thus, rDNA hyperthermic-induced hypercondensation is a specialized and unique response that is independent of most heat shock pathways. Of particular interest, the results reveal that neither deletion of the Sir2 (NAD+ deacetylase and major regulator of rDNA silencing and structure) nor Fob1 (the rDNA replication fork barrier protein that coordinates rDNA replication with transcription) had any adverse impact on rDNA hyperthermic-induced hypercondensation (Figure 3B) (Gottlieb and Esposito 1989; Fritze *et al.* 1997; Smith and Boeke 1997; Imai *et al.* 2000; Machín *et al.* 2004; Johzuka and Horiuchi 2009; Kobayashi and Sasaki 2017). These results highlight the physiological separation of these pathways from rDNA hypercondensation. Notably, *top1* null cells exhibited 44% of puff-like rDNA structures even at 23° (similar condensation defects were observed at 37°), indicating that the rDNA was decondensed regardless of temperature. Thus, *top1* was excluded from further analyses into the mechanism of hyperthermic-induced hypercondensation. Our results, however, document that Top1 is critical for rDNA condensation at all temperatures (Figure 3B), consistent with prior findings that *top1* promotes condensation in *Drosophila melanogaster* and that *top1* null cells exhibit rDNA condensation defects in budding yeast (Castaño *et al.* 1996; Zhang *et al.* 2000).

### Hsp90 mutants are defective in hyperthermic-induced rDNA hypercondensation

**Figure 4:**
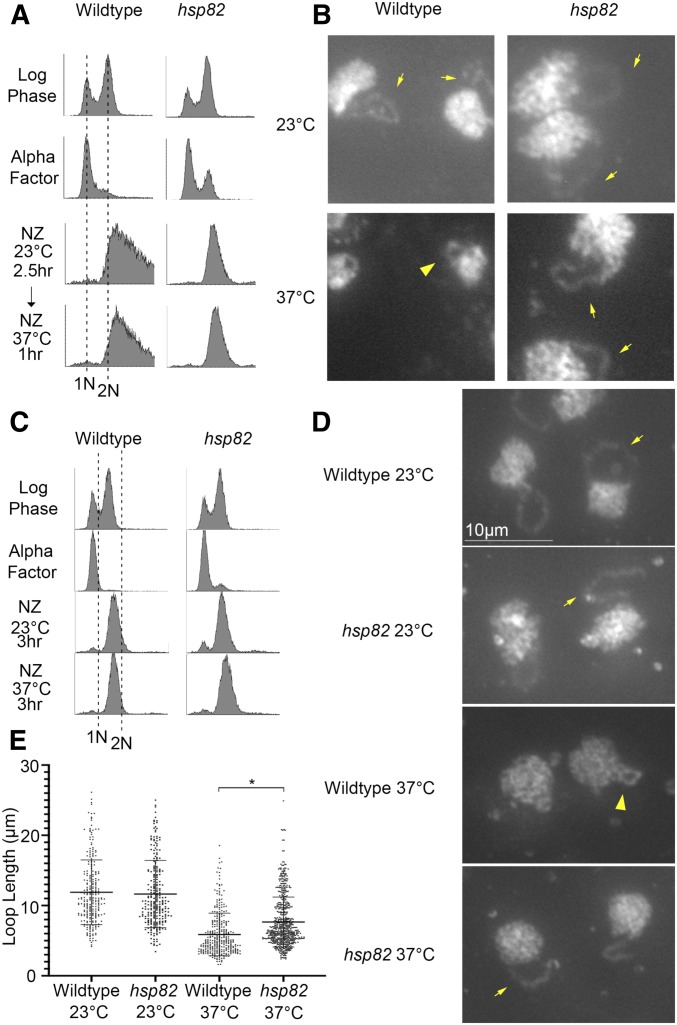
Hsp82 promotes hyperthermic-induced rDNA hypercondensation. (A) Flow cytometer data documents DNA content for wildtype and *hsp82* synchronization (note that wildtype DNA profiles also appear in Figure 3A). Cells were maintained in nocodazole for 2.5 hr at 23°, postalpha factor synchronization/release, followed by an additional 1 hr incubation at 37°. (B) Chromosomal mass and rDNA loop structures, detected using DAPI, from cells obtained following a 1 hr shift up to 37°. Yellow arrows indicate long rDNA loops. Yellow arrowheads indicate short rDNA loops. (C) Flow cytometer data of DNA content for wildtype and *hsp82* synchronization. Cells were maintained in nocodazole for 3 hr at 23° or 37°, post alpha factor synchronization/release. (D) Chromosomal mass and rDNA loop structures, detected using DAPI, from cells obtained following a 3 hr incubation at 37°. Yellow arrows indicate long rDNA loops. Yellow arrowheads indicate short rDNA loops. (E) Quantification of the loop lengths of condensed rDNA in wildtype (YPH499) and *hsp82* null mutant (YDS203) cells. Data obtained from three biological replicates in which *n* values are 251 for wild-type cells at 23°, 254 for *hsp82* cells at 23°, 323 for wild-type cells at 37°, 340 for *hsp82* cells at 37°. Error bars represent SD of each sample. Statistical analyses were performed using Tukey HSD one way ANOVA. *P*-value = 0.001 indicates significant differences between the average loop lengths of wild-type cells (5.88 μm) *vs.* the *hsp82* mutant cells (7.65 μm) at 37°. Statistically significant differences (*) are based on *P* < 0.05. All micrographs are shown at the same magnification (see Bar in D).

Given the basis of our bioinformatics-based strategy, we were surprised to find an HSP/C that indeed impacts hyperthermic-induced rDNA condensation. Condensation assays in which hypercondensation was induced for a single hour during a mitotic arrest revealed that *hsp82* null cells fully support normal rDNA condensation during mitosis at 23°, but failed to completely hypercondense rDNA to wildtype levels in response to 37° incubation (Figure 4, A and B). To both extend and quantify the extent of this rDNA hypercondensation defect, we synchronized wild-type and *hsp82* deletion cells in G1 at 23°, then released divided cultures into either 23° or 37° medium supplemented with nocodazole to arrest cells in preanaphase. Cell cycle progression and arrests were monitored using flow cytometry (Figure 4C). We then measured the axial rDNA loop length from three biological replicates. The results reveal that mitotic *hsp82* mutant cells shifted to 37° contain significantly longer (roughly 30%) rDNA loops than wild-type cells shifted to 37° (Figure 4, D and E). Importantly, both wild-type and *hsp82* deletion cells exhibited similarly long loops at 23°, further highlighting the unique role for Hsp82 in specifically driving hyperthermic-induced rDNA hypercondensation.

**Figure 5:**
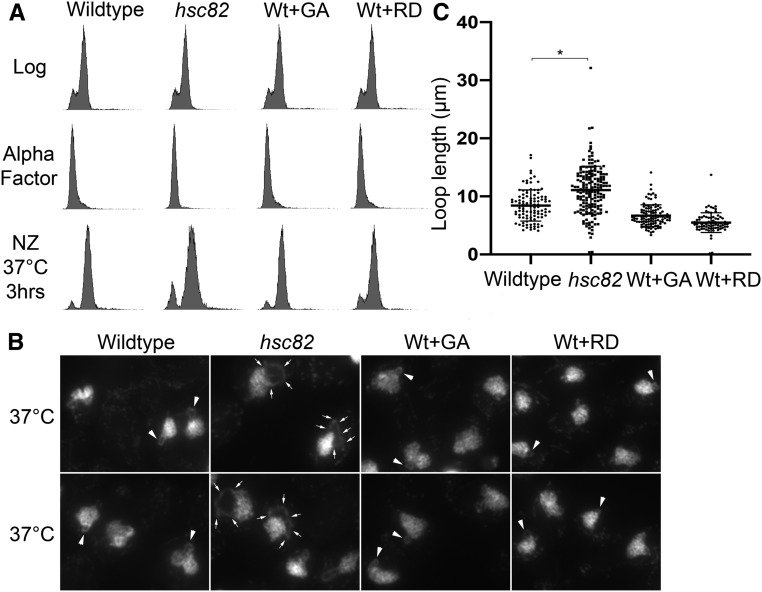
Impact of Hsc82 deletion and Hsp90 inhibitors on hyperthermic-induced rDNA hypercondensation. (A) Flow cytometer data documents DNA content for wild-type (YPH499) and *hsc82* null cells (and see Figure S3). For each, log phase cells were synchronized in G1 using alpha factor, then released into 37° fresh medium supplemented with nocodazole (NZ) for 3 hr to arrest cells in preanaphase. Note that the culture of G1 synchronized wildtype cells was split into three aliquots [NZ+/− Geldanamycin (GA) or Radicicol (RD)], which is reflected by duplication of the Log and G1 DNA profiles for those treatments. (B) Chromosomal masses and rDNA loop structures were detected using DAPI. White arrows indicate the track of rDNA long loops, arrowheads indicate rDNA short loops. (C) Quantification of loop lengths of condensed rDNA in wildtype (YPH499) and *hsc82* null cells (YDS210). The quantified results are based on *n* values of 102 for wild-type cells at 37° and 170 for *hsc82* cells at 37°. Error bars represent SD of each sample. Statistical analyses were performed using Tukey HSD one way ANOVA. *P*-value = 0.001 indicates significant differences between the average loop lengths of wild-type cells (4.21 μm) *vs.* the *hsc82* mutant cells (5.53 μm) at 37°. Statistically significant differences (*) are based on *P* < 0.05.

In yeast, Hsp90 family members include paralogs Hsp82 and Hsc82 that are 97% identical at the amino acid level, but exhibit differences in their expression (Kravats *et al.* 2018). Thus, it became important to determine the extent to which *hsc82* null cells phenocopy the hyperthermic-induced rDNA hypercondensation defect observed in *hsp82* null cells. Wildtype and *hsc82* deletion cells were synchronized in G1 at 23° using alpha factor, then released into 37° fresh medium supplemented with nocodazole for 3 hr to arrest cells in preanaphase as described above. Cell cycle progression and arrests were monitored using flow cytometry (Figure 5A). The results reveal that *hsc82* null cells exhibit a 40% increase in rDNA loop length, compared with wild-type cells (Figure 5, B and C). A statistically significant increase (30%) in rDNA loop lengths was also observed in a second iteration, in which we compared *hsc82* null cell rDNA loops lengths to those obtained from a BY4741 background strain (Figure S3). In combination, our combined findings from both *hsc82* and *hsp82* null cells, compared with eight other heat-response proteins, reveal that Hsp90 function is required for thermic-induced rDNA hypercondensation.

Hsp90 family members exhibit receptor/kinase signal transduction activities and are well-established ATP-dependent foldases that promote protein maturation and thermic tolerance by ensuring proper folding of client proteins (Khurana *et al.* 2018; Morán Luengo *et al.* 2019; Genest *et al.* 2019). In addition, however, Hsp90 family members also exhibit “holdase” functions independent of ATP binding/hydrolysis that include structural or scaffolding roles (Csermely *et al.* 1998; Hoter *et al.* 2018; Genest *et al.* 2019). In this light, we were particularly intrigued by early electron microscopy (EM) studies through which Hsp90 was localized to the nucleolus and onto chromatin fibrils (Ohtani *et al.* 1995; Biggiogera *et al.* 1996). We thus decided to test whether inhibition of Hsp90 ATPase activity, while retaining Hsp90 scaffolding function, would adversely impact hyperthermic-induced rDNA hypercondensation. Geldanamycin (GA) and Radicicol (RD) are both potent Hsp90 inhibitors that bind the ATP binding pocket and preclude foldase activity (Roe *et al.* 1999; Wider *et al.* 2009; Chen *et al.* 2012; Theodoraki *et al.* 2012; Millson and Piper 2014). Wild-type cells were synchronized in G1, using alpha factor, and then released into 37° rich medium supplemented with nocodazole alone or further supplemented with either GA (40 µM) or RD (20 µM *vs.* 40 µM for two experimental iterations) for 3 hr to arrest cells in preanaphase in the absence of Hsp90 ATPase activity. Cell cycle progression and arrests in this shift-up experiment were confirmed using flow cytometry (Figure 5A and Figure S3A). We then measured axial rDNA loop lengths from two biological replicates in which each contains at least 100 cells. Neither Hsp90 inhibitor (GA or RD) had any adverse effect on rDNA hypercondensation (Figure 5, B and C and Figure S3, B and C). GA and RD entry into yeast and inhibition of Hsp90 are likely immediate with overt responses obtainable within minutes (Tahbaz *et al.* 2001; Theodoraki *et al.* 2012). We validated GA-dependent Hsp90 inhibition on the Hsp90 client protein Chl1, a DNA helicase that promotes Scc2/cohesin deposition onto DNA (Figure S4; Tahbaz *et al.* 2001; Skibbens 2004; Khurana *et al.* 2018). In combination, these results provide intriguing evidence that Hsp90 proteins may play ATP-independent “holding” activities that are critical for rDNA responses to thermic stress.

### Hmo1 negatively regulates mitotic rDNA condensation

During our screen of 10 HSP/C null cells function in hyperthermic-induced rDNA hypercondensation, we observed that *hmo1* null cells contained two distinct condensed rDNA loops—often appearing as rabbit ears (Figure S5A). The fact that isolates from this strain had diploidized was confirmed by flow cytometry (Figure S5B). Intriguingly, these *hmo1* null cells often failed to arrest with a 2N DNA content in response to medium supplemented with nocodazole (Figure S5B), suggesting that there are additional mutations that reside in the *hmo1* deletion strain (Winzeler *et al.* 1999; Giaever and Nislow 2014; VanderSluis *et al.* 2014). Regardless of the aforementioned phenotypes, we noted that *hmo1* isolates also contained aberrant rDNA loop lengths (see below).

**Figure 6:**
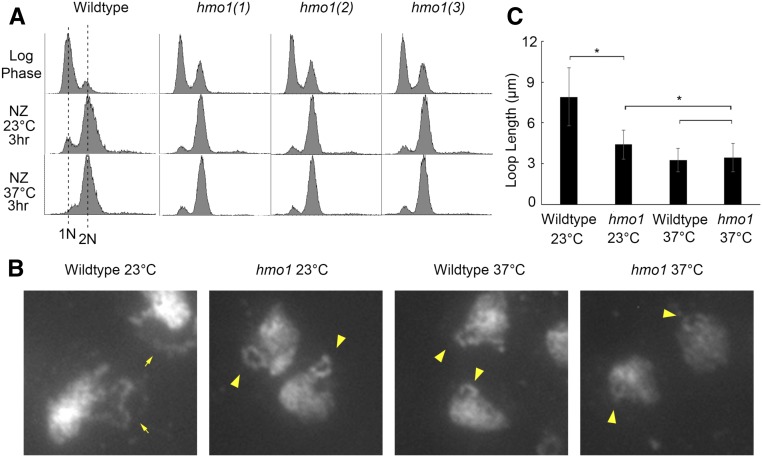
Hmo1 negatively regulates mitotic rDNA condensation. (A) Flow cytometer data documents DNA content throughout the experiment. Log phase cultures were split and equal portions incubated for 3 hr at either 23° or 37° in fresh media supplemented with nocodazole. (B) Chromosomal mass and rDNA loop structures detected using DAPI. Yellow arrows indicate long rDNA loops. Yellow arrowheads indicate short rDNA loops. (C) Quantification of the loop lengths of condensed rDNA in wildtype (YPH499) and three *hmo1* deletion mutant (YBS3047, YBS3048, YBS3049) cells. Graphed values are based on *n* values of 100 for Wildtype cells at 23°, 182 for *hmo1* cells at 23°, 48 for Wildtype cells at 37°, and 159 for *hmo1* cells at 37°. Error bars represent SD of each sample. Statistical analysis performed using Tukey HSD one way ANOVA. *P*-value = 0.001 indicates there is significant differences between the average loop lengths of wildtype cells *vs.* the *hmo1* mutant cells arrested at 23°. *P*-value = 0.001 indicates there is significant differences between the average loop lengths of *hmo1* mutant cells arrested at 23° *vs.* 37°. *P*-value = 0.756 indicates there is no significant differences between the average loop lengths of wildtype cells (3.24 μm) *vs.* the *hmo1* mutant cells (3.44 μm) arrested at 37°. Statistically significant differences (*) are based on *P* < 0.05.

To further investigate Hmo1 function in hyperthermic-induced rDNA hypercondensation, we generated new *hmo1* null strains in the S288C wildtype background, confirming specific gene replacement by PCR (see *Materials and Methods*). We then synchronized wildtype and three independent *hmo1* null isolates for 3 hr (at 23° or 37°) in medium supplemented with nocodazole, and then measured rDNA loop lengths for each of the preanaphase-arrested strain. Flow cytometry results document that each of our *hmo1* deletion isolates is haploid and arrests in response to nocodazole (Figure 6A). As expected, wild-type cells contained extended rDNA loops at 23° and hypercondensed rDNA loops at 37° (Figure 6B). Surprisingly, *hmo1* mutant cells contained significantly shorter loops at 23°, compared with wild type. Upon exposure to 37°, however, the rDNA loops in *hmo1* null cells hypercondensed to a length similar to that exhibited by wild-type cells (Figure 6, B and C). Thus, while mitotic *hmo1* cells contain increased levels of rDNA condensation at 23°, a shift to 37° does not promote rDNA hypercondensation beyond that observed in wild-type cells. Given that *hmo1* null cells exhibit elevated rDNA condensation in the absence of hyperthermic stress, we term Hmo1 a novel negative regulator of mitotic rDNA condensation.

## Discussion

The nucleolus and rDNA are exquisitely tuned to both the cell cycle and external cues. For instance, rDNA prematurely condenses during interphase in response to starvation and also condenses in a stereotypic fashion during each entry of the cell into mitosis (Guacci *et al.* 1994; Li *et al.* 2006; Tsang *et al.* 2007). All of these structural changes require condensins with an additional role played by cohesins during mitotic condensation (Skibbens *et al.* 1999; Tóth *et al.* 1999; Guacci *et al.* 2004; Kueng *et al.* 2006; Tsang *et al.* 2007; Hirano 2012; Lopez-Serra *et al.* 2013; Wang *et al.* 2016). Recently, we and others identified a novel form of hypercondensation that occurs during mitosis in response to heat stress, and, thus far, appears specific to the rDNA (Shen and Skibbens 2017a; Matos-Perdomo and Machín 2018a). The first major finding of the current study is that rDNA hyperthermic-induced hypercondensation occurs independently of condensin recruitment/dissociation or inactivation of factors that regulate cohesin binding/dissociation. This surprising result suggests that the last several decades of research into rDNA structure analyses remain narrowly focused on SMC complexes, and that our understanding of chromatin structure regulation remains incomplete.

Recently, a condensation end-state (also referred to as hypercondensation) that required additional condensin recruitment was reported to occur during anaphase (de los Santos-Velazquez *et al.* 2017). A second major finding of the current study, based on analyses of anaphase promoting complex mutant strains, is that this normal chromatin condensation end-state reported by de los Santos-Velazquez and colleagues is distinct from the thermic-induced rDNA hypercondensation that occurs during preanaphase. Moreover, new findings reported here document that the significant rDNA loop shortening that occurs during preanaphase proceeds in the absence of additional condensin recruitment. In support of these distinct mechanisms, we note that thermic-induced rDNA hypercondensation is rapidly reversible, while the proteolytic mechanism that underlies anaphase onset is not (Amon *et al.* 1994; Cohen-Fix *et al.* 1996; Uhlmann *et al.* 1999; Shen and Skibbens 2017a).

A third major finding of the current study is that mutation in either *HSP82* and *HSC82*, which encode members of the Hsp90 HSP/C family, results in defective hyperthermic-induced rDNA hypercondensation. Here, we consider several possibilities regarding the mechanism through which HSP/C impact thermic-induced rDNA hypercondensation. Given the well-established role for HSP/C in stabilizing or refolding client proteins (Khurana *et al.* 2018; Genest *et al.* 2019; Morán Luengo *et al.* 2019), one plausible mode of action is through stabilization of an, as yet undefined, thermic-sensitive client protein required for rDNA hypercondensation. This client is unlikely to include cohesin or condensin, given that thermic stresses in HSP/C mutant cells result in increased rDNA axial loop lengths but not loss of either loop morphology (loops transitioning to puffs) or sister chromatid cohesion (one loop transitioning to two loops) (Shen and Skibbens 2017a; Matos-Perdomo and Machín 2018a). In contrast, cohesin inactivation quickly results in rDNA puff structures and cohesion loss (Guacci *et al.* 1994, 1997; Lavoie *et al.* 2002; Lavoie *et al.* 2004; D’Ambrosio *et al.* 2008; Lopez-Serra *et al.* 2013; Tong and Skibbens 2015; Shen and Skibbens 2017a). We further note that thermic-induced hypercondensation appears to effect rDNA specifically (Shen and Skibbens 2017a), while cohesins and condensin impact chromatin architecture genome-wide (Mayer *et al.* 2004; Skibbens 2004; Xu *et al.* 2007; Rudra and Skibbens 2012; Borges *et al.* 2013; Samora *et al.* 2016; Shen and Skibbens 2017b). Importantly, potent Hsp90 ATP-binding/hydrolysis inhibitors GA and RD (Roe *et al.* 1999; Wider *et al.* 2009; Chen *et al.* 2012; Theodoraki *et al.* 2012; Millson and Piper 2014) both failed to adversely impact hyperthermic-induced rDNA condensation (current study). Thus, a novel and exciting possibility is that Hsp90 family members play a direct structural role in hypercondensing rDNA in response to heat stress. This model is supported both by EM studies that localize Hsp90 to the nucleolus, and also by circular dichroism spectra studies that Hsp90 induces *in vitro* a more condensed chromatin state in rat liver cells (Csermely *et al.* 1998; Ohtani *et al.* 1995; Biggiogera *et al.* 1996). A third possibility is that Hsp90 “holdases” regulate factors that in turn promote rDNA hypercondensation. For instance, Hsp82 exhibits synthetic growth defects with histone (H2B), histone variant (H2A.Z), histone modifiers, and chromatin remodeling complexes (Dep1, Eaf1,7, Gcn5, Gis1, Hda2,3, Pho23, Rco1, Rtt109, Sap30, Set2, and Swi3) (Millson *et al.* 2005; Zhao *et al.* 2005; McClellan *et al.* 2007). Such histone modification cascades (including deacetylation, phosphorylation, and tail–tail interaction of adjacent histones) may promote rDNA hypercondensation during mitosis (Wilkins *et al.* 2014). Future efforts are required to resolve the issue of whether Hsp82 and Hsc82 directly impose rDNA structure or promote (through foldase or holdase activities) other factors to induce hyperthermic-induced rDNA hypercondensation.

**Figure 7:**
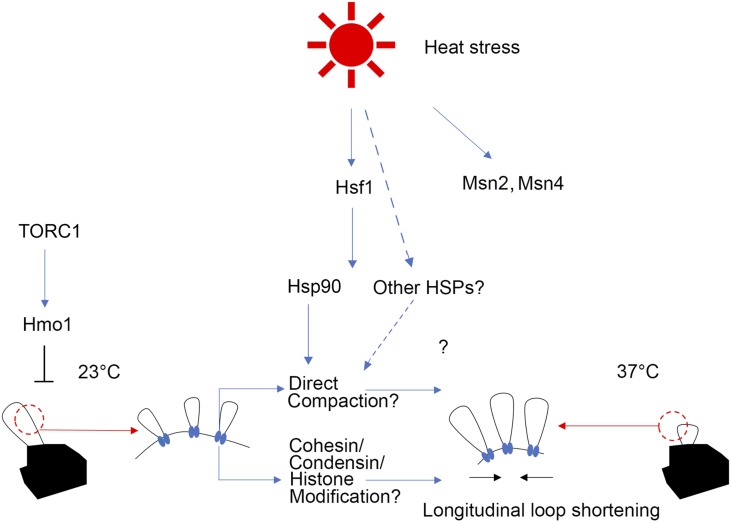
Possible mechanisms of Hsp90-dependent hyperthermic-induced rDNA hypercondensation. Hsp90 functions in hyperthermic-induced rDNA hypercondensation possibly through (1) direct interaction of rDNA; (2) recruitment of enzymes that post-translationally modify cohesins, condensins, or histones; or (3) by stabilizing client proteins that perform novel roles in rDNA structure (data not shown). Here, we suggest that Hmo1 may be involved in TORC1 signaling that antagonize rDNA condensation. Hmo1 inhibits hyperthermic-induced rDNA hypercondensation such that rDNA hypercondenses in the absence of Hmo1. In the schematic (23° on the left, 37° on the right), large black objects indicate chromatin mass from which an rDNA loop (thin black lines) protrudes. Red dotted circles indicate a magnified view of the rDNA. Blue bases are putative rDNA hypercondensation factors. The transition in which some portion of short longitudinal rDNA loops at 23° give rise to the formation of lateral rDNA loops (orthogonal to the longitudinal axis) at 37°, to generate transcriptionally active rDNA segments, is hypothetical, but consistent with the observation that cells growth rates are increased at 37°, indicating increased transcription.

While the results presented here argue against a role for either cohesin or condensin deposition/release in driving rDNA hypercondensation, we cannot rule out a model in which post-translational modifications alter the condensing activities of these SMC complexes. For instance, it is well established that condensin phosphorylation promotes mitotic condensation (Kinoshita and Hirano 2017; Kalitsis *et al.* 2017; Kakui and Uhlmann 2018). Cdc28 is the cyclin-dependent kinase that phosphorylates condensin and triggers condensation during prophase (Hadwiger *et al.* 1989; Surana *et al.* 1991). It is thus notable that Hsp82 and Cdc28 physically interact (Zarzov *et al.* 1997), providing a mechanism through which Hsp82-recuitment of CDK might activate condensins without altering deposition/release dynamics. Intriguingly, Cdc14 phosphatase also impacts rDNA condensation and physically interacts with Hsp82, potentially revealing a complex interplay between post-translational modification of condensins and rDNA architecture prior to anaphase (Sullivan *et al.* 2004; Woodford *et al.* 2016; de los Santos-Velazquez *et al.* 2017). Cohesin modification, in the absence of deposition/dissociation, might also contribute to rDNA hypercondensation upon thermic stress during preanaphase. For instance, *hsp82* mutants exhibit synthetic growth defects in combination with mutation of the cohesin acetyltransferase *ECO1* (Skibbens *et al.* 1999; Tóth *et al.* 1999; Millson *et al.* 2005; Rolef Ben-Shahar *et al.* 2008; Borges *et al.* 2013). While there is a paucity of evidence that physically links Eco1 to Hsp82, it remains formally possible that Hsp82 promotes rDNA hypercondensation through Eco1 acetylation of cohesin subunits. In contrast to reports that Hsp82 promotes sister chromatid cohesion through stabilization of Chl1 DNA helicase (Khurana *et al.* 2018), which, in turn, promotes both Scc2 and cohesin binding to DNA (Mayer *et al.* 2004; Skibbens 2004; Xu *et al.* 2007; Rudra and Skibbens 2012; Borges *et al.* 2013; Samora *et al.* 2016; Shen and Skibbens 2017b), we find no evidence in the current study that Chl1 (or changes in Scc2-mediated cohesion or direct condensin deposition) adversely impacts rDNA hypercondensation. Future studies will be required to ascertain the extent to which histone, cohesin, and/or condensin modifications promote hyperthermic-induced rDNA hypercondensation in an Hsp90-dependent manner (Figure 7).

A fourth major finding of the current study is that the High Mobility Group protein Hmo1, which is involved in TOR signaling, negatively regulates mitotic rDNA condensation. Inhibition of TOR by rapamycin causes nucleolar contraction, condensin loading on to rDNA during interphase, and also promotes rDNA hypercondensation in both pre-anaphase and anaphase-arrested cells (Tsang *et al.* 2007; de los Santos-Velazquez *et al.* 2017; Matos-Perdomo and Machín 2018a,b). Thus, TOR inhibition might trigger increased condensation of rDNA through Hmo1. Despite the increased condensation state of rDNA that occurs at 23° in *hmo1* deletion cells, rDNA remains competent to hypercondense upon a temperature shift to 37°. This latter hypercondensed state is similar to that exhibited by wildtype cells at 37°, suggesting that Hmo1 antagonizes rDNA compaction through a mechanism separate from Hsp90-dependent hyperthermic-induced rDNA hypercondensation. Conversely, Hmo1 promotes rDNA transcription and triggers DNA bridging and looping that contributes to higher-order architectures (Albert *et al.* 2013; Divakaran *et al.* 2014; Murugesapillai *et al.* 2014; Wang *et al.* 2016). Thus, on the one hand, Hmo1 promotes condensation (in a condensin-dependent fashion) during interphase in response to nutrient starvation while, on the other hand, antagonizes mitotic condensation (Wang *et al.* 2016 and the current study).

It is remarkable that a GO-term based bio-informatic screen, in which we limited analyses to only 10 candidates, turned up two factors: Hsp82, which is critical for hyperthermic-induced rDNA hypercondensation; and Hmo1, which negatively regulates canonical mitotic condensation. We were able to validate our results for the role of Hsp82 by exploiting the highly conserved paralog Hsc82. While the mechanism through which HSP/C impacts thermic-induced rDNA hypercondensation remains unknown, this is the first report that HSP/C support this process and suggest that future endeavors will continue to uncover novel roles for HSP/C function in chromatin structure. Our results further document that single mutant *hsp82* and *hsc82* null cells are only partially inhibited for hyperthermic-induced rDNA hypercondensation. Synthetic lethality and/or severe growth defects precluded testing *hsp82* *hsc82* double mutants (McClellan *et al.* 2007; Hainzl *et al.* 2009). Given that >1000 genes are involved in the heat shock response in budding yeast (Morano *et al.* 2012), we anticipate that the identification of redundant pathways will uncover novel mechanisms through which HSP/C factors help promote alterations in chromatin structure.
